# Hyperoside pre-treatment prevents glomerular basement membrane damage in diabetic nephropathy by inhibiting podocyte heparanase expression

**DOI:** 10.1038/s41598-017-06844-2

**Published:** 2017-07-25

**Authors:** Xiaofei An, Lin Zhang, Yanggang Yuan, Bin Wang, Qiuming Yao, Ling Li, Jisheng Zhang, Ming He, Jinan Zhang

**Affiliations:** 1 Department of Endocrinology, Jinshan Hospital of Fudan University, Shanghai, 201508 China; 2 Department of Biochemistry and Molecular Cell Biology, Shanghai Jiao Tong University School of Medicine (SJTU-SM), Shanghai, 200025 China; 3 Department of Nephrology, The First Affiliated Hospital of Nanjing Medical University, Jiangsu Province People’s Hospital, Nanjing, 210029 China; 4 Department of Otorhinolaryngology, Affiliated Hospital of Qingdao University, Qingdao, 266003 China; 5 Department of Pathophysiology, Key Laboratory of Cell Differentiation and Apoptosis of Chinese Ministry of Education, Shanghai Jiao Tong University School of Medicine (SJTU-SM), Shanghai, 200025 China

## Abstract

Glomerular basement membrane (GBM) damage plays a pivotal role in pathogenesis of albuminuria in diabetic nephropathy (DN). Heparan sulfate (HS) degradation induced by podocyte heparanase is the major cause of GBM thickening and abnormal perm-selectivity. In the present study, we aimed to examine the prophylactic effect of hyperoside on proteinuria development and GBM damage in DN mouse model and the cultured mouse podocytes. Pre-treatment with hyperoside (30 mg/kg/d) for four weeks could significantly decrease albuminuria, prevent GBM damage and oxidative stress in diabetes mellitus (DM) mice. Immunofluorescence staining, Real time PCR and Western blot analysis showed that decreased HS contents and increased heparanase expression in DN mice were also significantly improved by hyperoside pre-treatment. Meanwhile, transmission electron microscope imaging showed that hyperoside significantly alleviated GBM thickening in DN mice. In addition, hyperoside pre-treatment inhibited the increased heparanase gene (HPR1) promoter activity and heparanase expression induced by high glucose or reactive oxidative species (ROS) in cultured podocytes. Our data suggested that hyperoside has a prophylactic effect on proteinuria development and GBM damage in DM mice by decreasing podocyte heparanase expression.

## Introduction

Diabetic nephropathy (DN) is the most common pathologic disorder pre-disposing end-stage renal disease (ESRD)^1, 2^. The early stage of DN is clinically characterized with persistent micro-albuminuria and multiple renal pathological injuries^3^. Early morphological alterations in DN include glomerular hypertrophy, mesangial matrix expansion and GBM thickening^4, 5^. These can cause glomerular filtration barrier (GFB) dysfunction and then contribute to onset and development of micro-albuminuria^4, 6^.

GBM is regarded as the primary barrier to allow molecules in circulation to selectively across to urinary space^7–9^ and the previous studies have confirmed that GBM damage are the first and major step leading to proteinuria in DN^6, 10^. GBM consists mainly of laminin, type IV collagen, nidogen, and heparan sulfate (HS) proteoglycan^8, 9^ and HS is one of the major ingredients of GBM^7, 11^. Due to its negative charge, HS chains are highly hydrated and play a space-filling and molecular-sieving key role in GBM^11–13^. Although the direct role of HS in GBM filtration was challenged by several studies^14, 15^, HS is commonly accepted as a crucial determinant of GBM perm-selectivity which receives more attention in recent years^10, 13, 16, 17^.

Heparanase is an identified endo-β(1–4)-D-glucuronidase which can specifically cleave carbohydrate chains of HS in extracellular matrix such as GBM and glomerular endothelial glycocalyx^17–19^. Loss of HS contents degraded by heparanase in the GBM has been confirmed in experimental DN models and DN patients^19–21^. Heparanase-driven inflammatory cascade also plays an important role in pathogenesis of DN^22^. Renal heparanase, mainly in podocytes is abnormally up-regulated by high glucose, ROS and renin-angiotensin system (RAS) in diabetes condition^23–25^. The recent studies suggest that heparanase plays a crucial role in pathogenesis in DN^19, 26^.

Hyperoside is a flavone glycoside showing anti-oxidant, anti-cancer, and anti-inflammatory properties^27–29^. Hyperoside inhibited high glucose-induced vascular inflammations^27^ and mitigated cultured podocyte apoptosis induced by the advanced glycation end-products (AGEs)^30^. The recent study suggested that hyperoside could ameliorate glomerulosclerosis in DN by downregulating miR-21 and increasing matrix metalloproteinases-9 (MMP-9) expression^31^. Our previous study showed that hyperoside could decrease albuminuria at the early stage of DN through alleviating podocyte injury^32^. Based on the pathogenesis and prevalence of DN, it is clinically more important and meaningful to prevent the onset of albuminuria and GBM damage in DN than to cure them. Whether hyperoside has a prophylactic effect on development of albuminuria and can postpone the onset of DN in DM remains to be investigated.

The purpose of this study was to explore the possible prophylactic effect of hyperoside on development of albuminuria and GBM damage in STZ-induced DM mouse model. Furthermore, its effect on glomerular podocyte heparanase up-regulation *in vivo* and *in vitro* was also observed to clarify its molecular and pharmacologic mechanisms.

## Results

### Hyperoside pre-treatment alleviated albuminuria and ROS in DN mice

**Figure 1:**
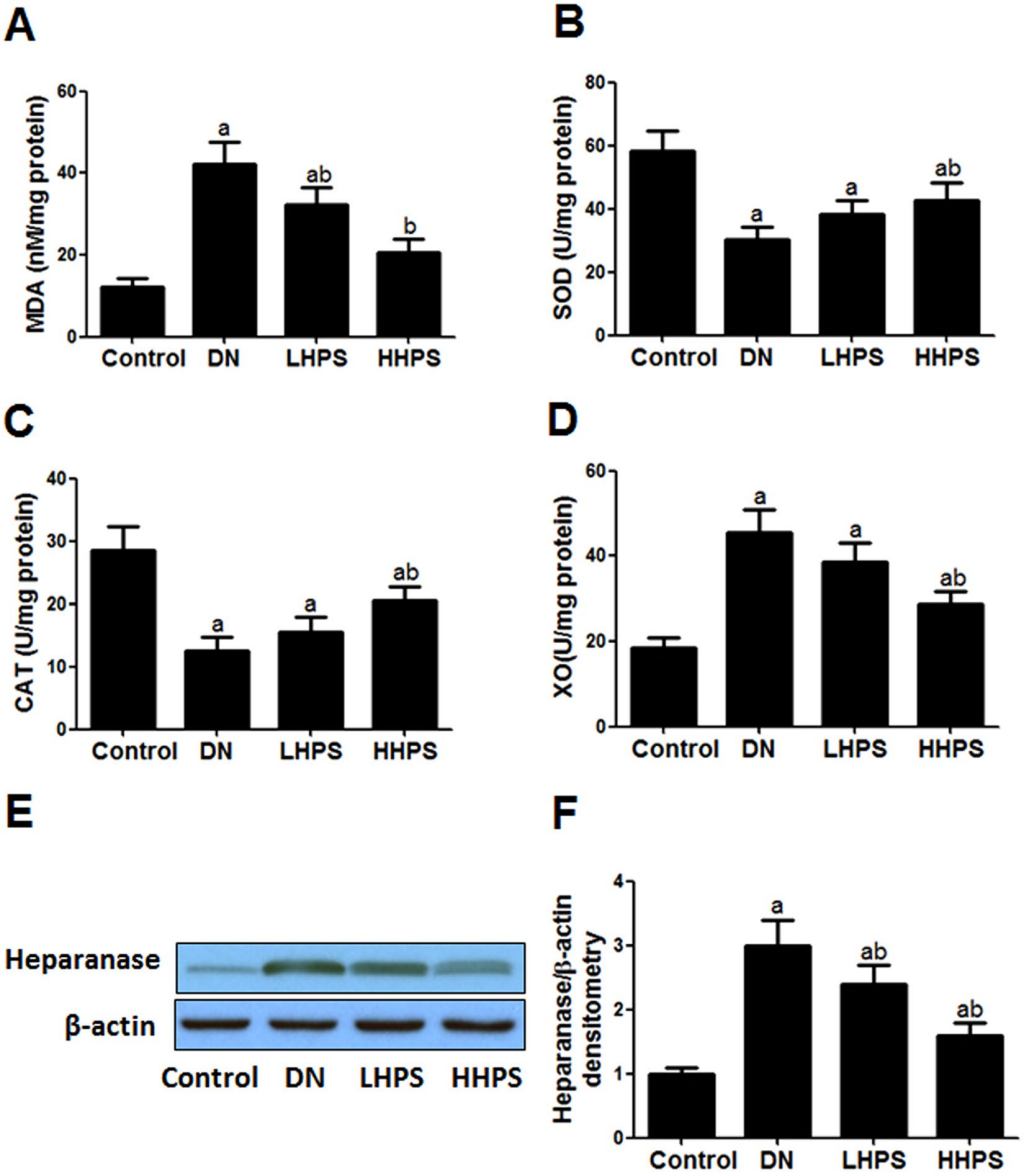
Hyperoside pre-treatment alleviated renal cortex oxidative stress and heparanase protein expression in DN mice (n = 15). (**A**) The levels of ROS-related end product MDA in renal cortex were measured among indicated groups. (**B**–**D**) The activity of ROS-related enzymes SOD, CAT and XO was measured among indicated groups. (**E**,**F**) Renal cortex heparanase protein expression was detected by western blot analysis and the columns showed the relative quatification of heparanase protein levels normalized to β-actin. Control: Normal mice; DN: DM mice were treated with vehicle solution for four weeks; LHPS and HHPS: DM mice were treated with 10 or 30 mg/kg/d hyperoside for four weeks respectively from two weeks after DM establishment. Data are presented as mean ± SD. P < 0.05 is statistically significant. ^a^Indicates significant vs. Control, ^b^indicates significant vs. DN.

To explore the prophylactic effect of hyperoside on albuminuria at the early stage DN mice, urinary mAlb/Cr was measured in the four groups. As compared with the normal mice, DN mice exhibited significant increase in urinary mAlb/Cr (P < 0.05). Pre-treatment with lower dose of hyperoside (10 mg/kg/d) had a decreasing effect on urinary mAlb/Cr but did not reach the statistically difference. Higher dose of hyperoside (30 mg/kg/d) for four weeks could significantly decrease the albuminuria in DN mice (P < 0.05). Both doses of hyperoside did not significantly affect blood pressure and glucose metabolism in DN mice (Table 1). The activities of ROS-related enzymes and ROS-related end product MDA in kidney reflect the status of renal oxidative stress. The MDA and ROS producing enzyme XO were significantly elevated in kidney cortex of DN mice by about three fold. Hyperoside pre-treatment (30 mg/kg/d) could significantly decrease the elevated MDA and XO levels (Fig. 1A,D). It could also increase the ROS preventing enzyme SOD and CAT levels which were found to be down-regulated in DN mice (Fig. 1B,C). Meanwhile, as the indicatives of ROS, increased MDA and decreased SOD levels in serum of DN mice were improved significantly by hyperoside pre-treatment (Table 1), indicating hyperoside displayed a decreasing effect on renal and serum ROS production in DN mice.

**Table 1:** The effect of hyperoside pre-treatment on proteinuria, glucose and lipid metabolism, blood pressure, serum RAS and ROS in DN mice (n = 15).

Index	Control	DN	LHPS	HHPS
UmAlb/Cr (mg/g)	16.5 ± 3.8	115.4 ± 14.2^a^	98.5 ± 10.5^a^	68.6 ± 7.3^a,b^
FBG (mmol/L)	5.7 ± 0.8	15.8 ± 2.8^a^	15.7 ± 2.4^a^	15.4 ± 3.3^a^
HbA1c (%)	5.6 ± 0.8	10.5 ± 1.5^a^	10.2 ± 1.4^a^	10.3 ± 1.2^a^
MAP (mmHg)	80.2 ± 5.8	82.6 ± 6.6	82.6 ± 5.4	82.3 ± 5.8
TC (mmol/L)	4.2 ± 0.5	4.9 ± 0.6	4.5 ± 0.7	4.6 ± 0.6
LDL (mmol/L)	2.3 ± 0.5	3.1 ± 0.6^a^	2.9 ± 0.5^a^	3.0 ± 0.5^a^
Ang II (pg/ml)	52.8 ± 7.3	68.3 ± 11.2^a^	66.4 ± 10.4^a^	62.7 ± 9.8^a^
Aldosterone (pg/ml)	108.4 ± 11.6	128.5 ± 10.4^a^	125.2 ± 11.6^a^	124.6 ± 13.5^a^
MDA (µmol/L)	3.2 ± 0.4	9.7 ± 1.5^a^	6.4 ± 0.8^a,b^	5.2 ± 0.7^a,b^
SOD (mU/L)	48.3 ± 6.5	22.3 ± 4.1^a^	34.5 ± 6.2^a,b^	40.6 ± 5.7^a,b^

Control: Normal mice; DN: DM mice were treated with vehicle solution; LHPS and HHPS: DM mice were treated with 10 or 30 mg/kg/d hyperoside for four weeks from two weeks after DM establishment respectively. Data are presented as mean ± SD. P < 0.05 is statistically significant. ^a^Indicates significant vs. Control, ^b^indicates significant vs. DN.

### Hyperoside pre-treatment decreased renal heparanase up-regulation in DN mice

**Figure 2:**
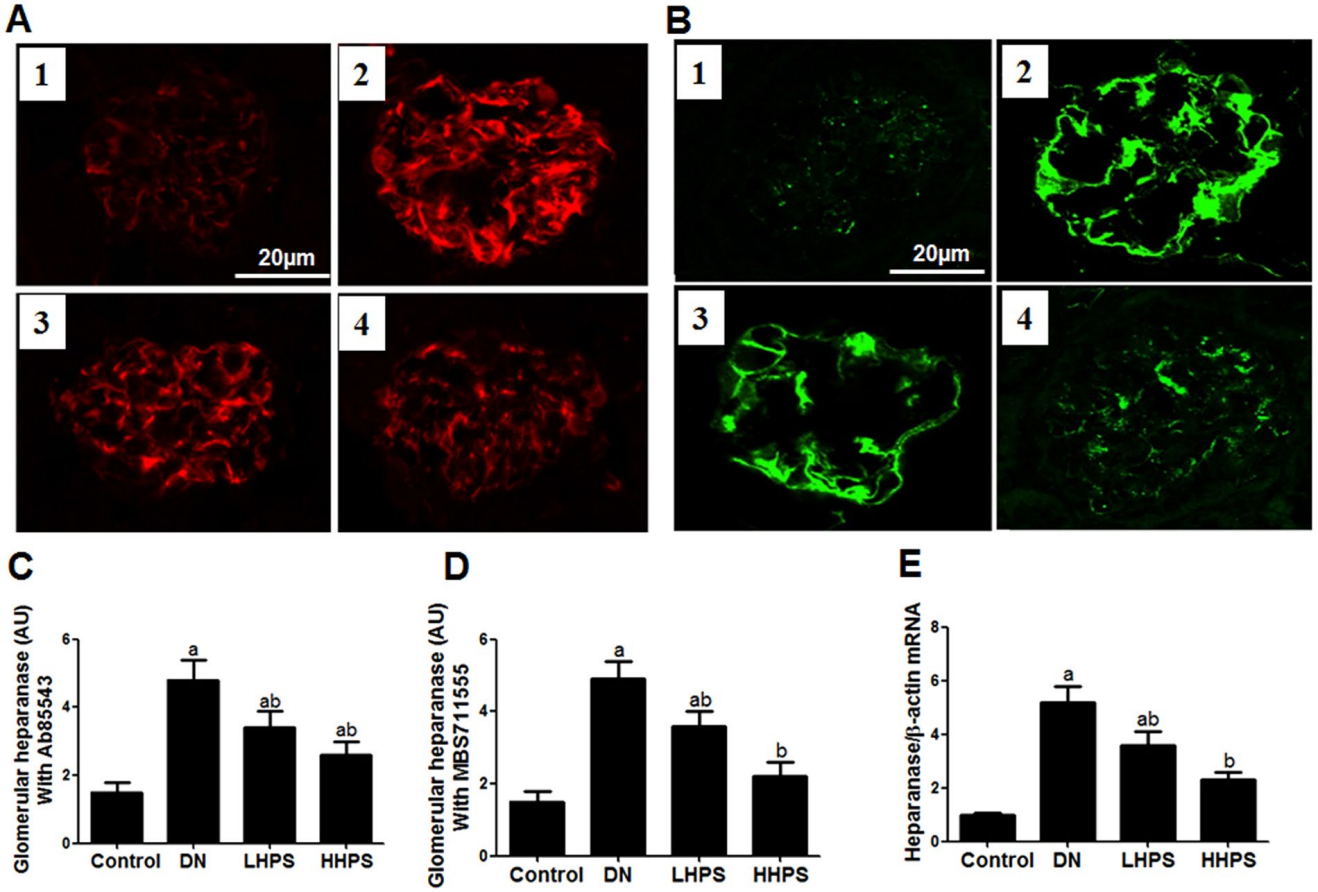
Hyperoside pre-treatment attenuated glomerular heparanase expression in DN mice (n = 5). (**A**) The glomerular heparanase expression was determined by immunofluorescence staining using the heparanase primary antibody (Ab85543) detecting 65 kD precursor as well as the 50 kD and 8 kD subunits of heparanase followed by rhodamine conjugated secondary antibody. (**B**) The heparanase primary antibody (MBS71555) followed by FITC conjugated secondary antibody was used to show 65 kD heparanase. 1:Control; 2:DN; 3:LHPS; 4:HHPS. (**C**,**D**) Quantification was performed by NIH Image software and shown in columns. (**E**) The columns showed the amount of heparanase mRNA levels relative to β-actin. Data are presented as mean ± SD. P < 0.05 is statistically significant. ^a^Indicates significant vs. Control, ^b^indicates significant vs. DN.

The immune-reactivity of heparanase was mainly distributed in mouse glomeruli and semi-quantitative analysis was adopted to measure the amount of glomerular heparanase positive area. As shown in immunofluorescence staining with two different heparanase primary antibodies, hyperoside pre-treatment (30 mg/kg/d) for four weeks could significantly inhibit the increased glomerular heparanase immune-reactivity in DN mice (Fig. 2A–D). Furthermore, quantitative PCR and western blot analysis showed that renal cortex heparanse expression was abnormally up-regulated in DN mice as compared with the normal mice (P < 0.05). Consistently, hyperoside pre-treatment could significantly decrease the up-regulated heparanase protein (Fig. 1E,F) and mRNA expression (Fig. 2E) in DN mice.

### Hyperoside pre-treatment ameliorated HS content loss and GBM thickening in DN mice

**Figure 3:**
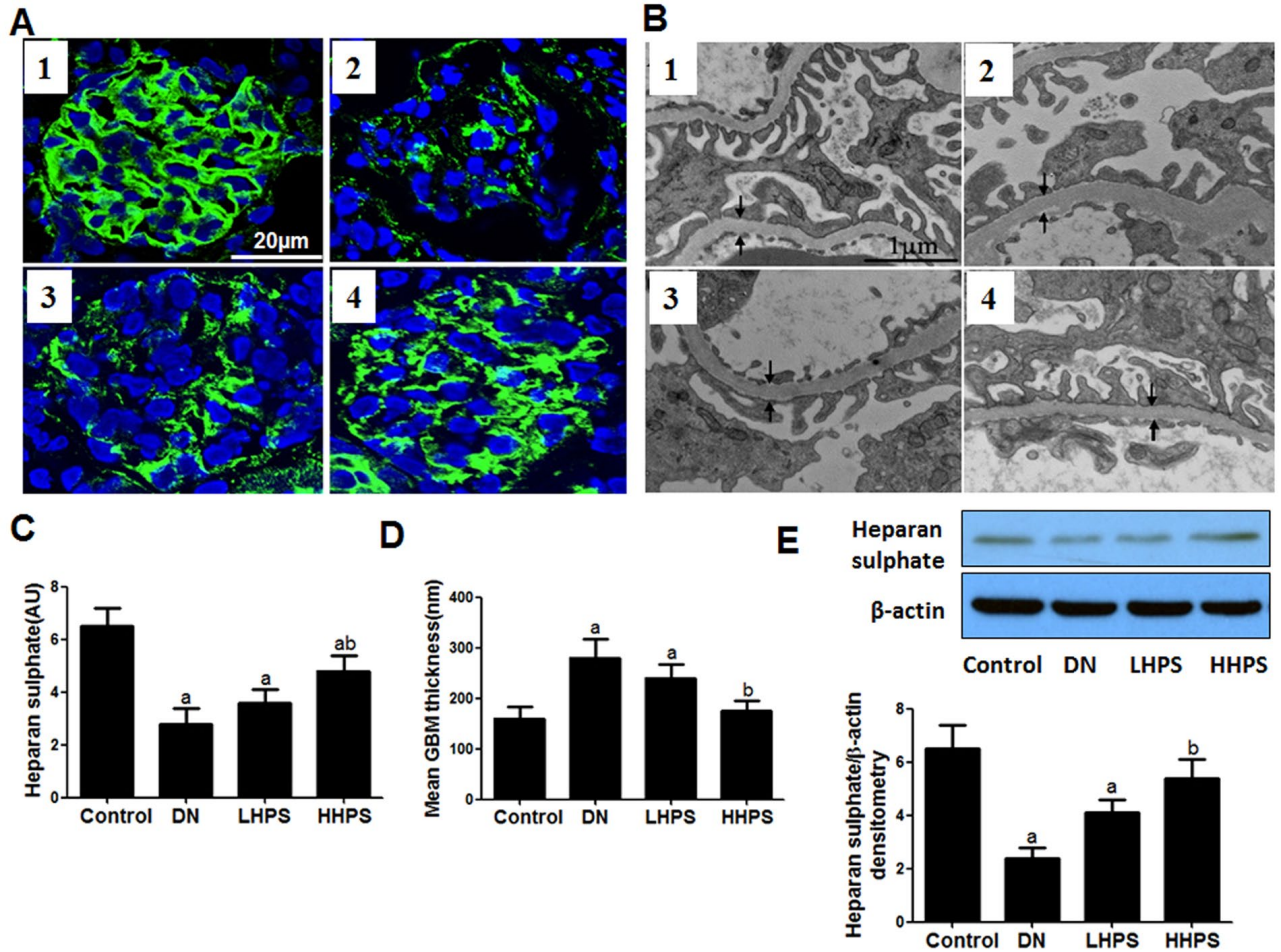
Hyperoside pre-treatment restored the decreased renal HS contents and attenuated GBM thickening in DN mice (n = 5). (**A**) The glomerular HS expression was determined by immunofluorescence staining. (**C**) The columns quantified the amount of immuno-reactive HS by NIH Image software. (**B**) GBM thickness was measured by transmission electron microscopy (×25000). (**D**) Quantifications of GBM thickness was presented in columns. (**E**) Western blot analysis of renal cortex HS protein levels and the columns showed the amount of HS protein levels relative to β-actin. Data are presented as mean ± SD. P < 0.05 is statistically significant. ^a^Indicates significant vs. Control, ^b^indicates significant vs. DN.

The immune-reactivity of HS protein was exclusively distributed in GBM and shown in a linear manner outside of DAPI positive area. Hyperoside pre-treatment (30 mg/kg/d) had a significant restoring effect on decreased glomerular HS immune-reactivity in DN mice (Fig. 3A,C). Accordingly, western blot analysis showed that hyperoside pre-treatment could markedly improve the decreased HS protein levels in renal cortex of DN mice (Fig. 3E). GBM thickness was assessed by transmission electron microscopy and semi-quantitative analysis^33^. The results indicated that hyperoside pre-treatment (30 mg/kg/d) for four weeks could significantly alleviate the degree of GBM thickening in DN mice (Fig. 3B,D).

### Hyperoside pre-treatment mitigated heparanase expression induced by high glucose

**Figure 4:**
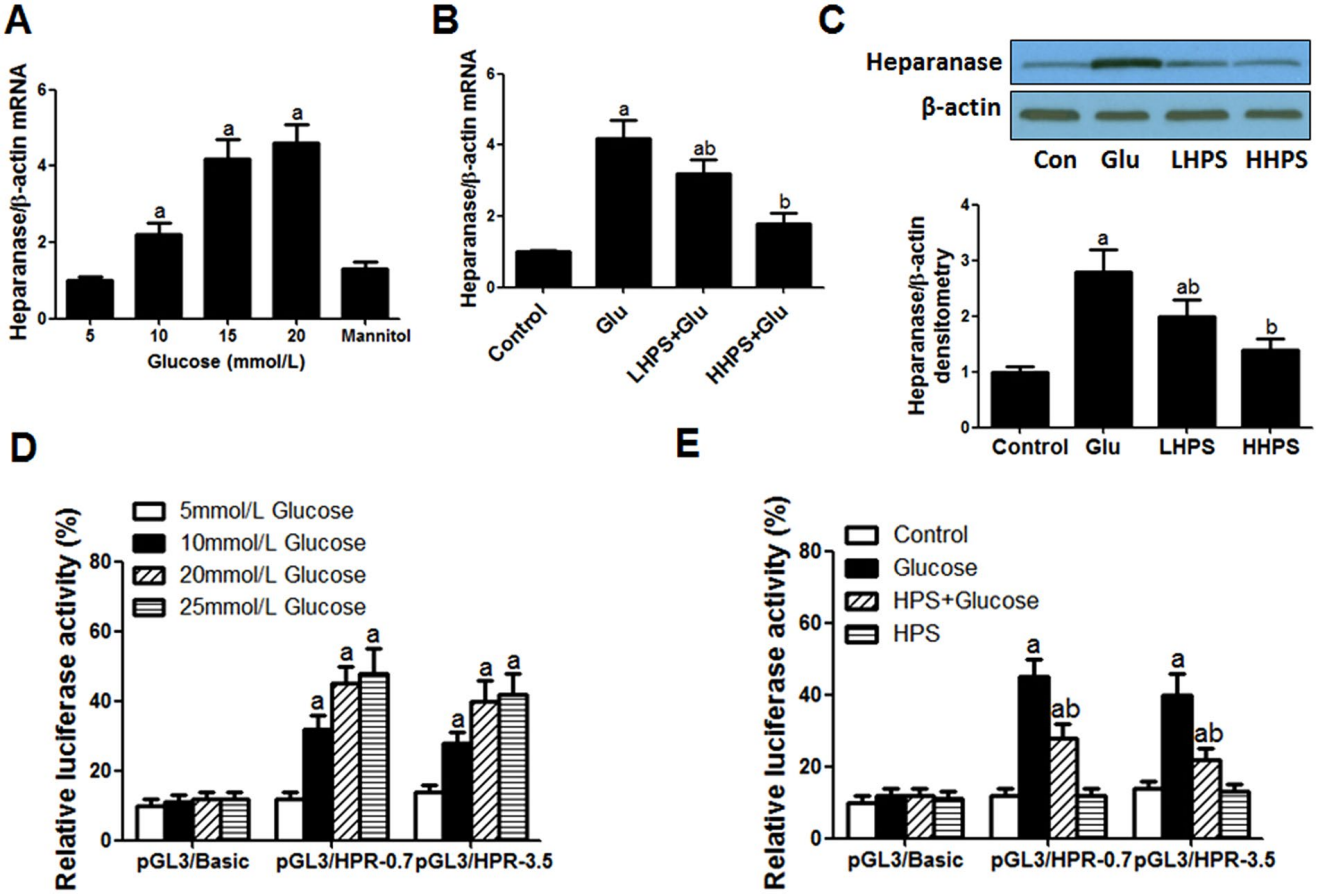
Hyperoside pre-treatment inhibited the increased heparanase expression and promoter activity induced by high glucose exposure in cultured podocytes. (**A**) The dose-response effect of glucose on podocyte heparanase mRNA expression. Cultured podocytes were incubated with 5 (as the control), 10, 15, 20 mmol/L glucose or mannitol of the same osmolity with 20 mmol/L glucose for 18 h. (**B**) Hyperoside pre-incubation attenuated the increased heparanase mRNA expression induced by 20 mmol/L glucose. Control: PBS incubation for 18 h; Glucose: 20 mmol/L glucose incubation for 18 h; LHPS or HHPS: 30 or 100 μg/ml hyperoside pre-incubation for 1 h and followed by 20 mmol/L glucose exposure. (**C**,**D**) Hyperoside pre-incubation attenuated heparanase protein expression induced by high glucose by western blot analysis. The columns showed the amount of heparanse protein levels relative to β-actin. Control: PBS incubation for 24 h; Glucose: 20 mmol/L glucose incubation for 24 h; LHPS or HHPS: 30 or 100 μg/ml hyperoside pre-incubation for 1 h and followed by 20 mmol/L glucose exposure. (**D**) The dose-related effect of glucose on heparanase promoter activity. Podocytes were grown under normal glucose conditions (5 mmol/L) and transfected with HPR1 promoter–driven luciferase reporter plasmid DNA or renilla luciferase gene as an internal control. After growing in complete medium containing 5, 10, 20 or 25 mmol/L glucose for 24 h, cells were harvested and measured for luciferase reporter activity. (**E**) Hyperoside pre-incubation attenuated increased heparanase promoter activity. Control: PBS incubation for 24 h; Glucose: 20 mmol/L glucose incubation for 24 h; HPS + Glucose: 100 μg/ml hyperoside pre-incubation for 1 h and followed by 20 mmol/L glucose exposure. HPS: 100 μg/ml hyperoside incubation alone. The relative light unit in each sample was normalized by renilla luciferase activity. Data are presented as mean ± SD. P < 0.05 is statistically significant. ^a^Indicates significant vs. Control, ^b^indicates significant vs. Glucose.

As compared with the 5 mmol/L glucose (as the Control), incubation of high concentration of glucose (10, 15 and 20 mmol/L) could up-regulate heparanase mRNA levels in cultured podocytes in a dose-related manner (Fig. 4A). The mannitol of the same osmolity with 20 mmol/L glucose had no significant effect on heparanase expression, which excluded the effect of high osmolity on heparanase expression. Pre-incubation with hyperoside (30 or 100 µg/ml) could significantly decrease the up-regulated heparanase mRNA levels induced by 20 mmol/L glucose (Fig. 4B). Accordingly, hyperoside pre-treatment could significantly decrease heparanase protein expression in cultured podocytes by western blot analysis (Fig. 4C).

### Hyperoside pre-treatment inhibited heparanase promoter activity induced by high glucose

To determine whether hyperoside could regulate HPR1 transcription induced by high glucose, a luciferase reporter assay was performed. The cultured podocytes was transfected with the luciferase reporter gene driven by a 0.7 or 3.5 kb HPR1 promoter. 10, 20 and 25 mmol/L glucose could induce luciferase activity by about four fold as compared with cells treated with 5 mmol/L glucose as the control (Fig. 4D). Pre-treatment with hyperoside (100 µg/ml) could significantly decrease the heparanase promoter activity induced by 20 mmol/L glucose (P < 0.05), whereas luciferase activity in cells with the empty vector was very low and not affected by glucose or hyperoside treatment (Fig. 4E).

### Hyperoside pre-treatment attenuated heparanase up-regulation induced by ROS

**Figure 5:**
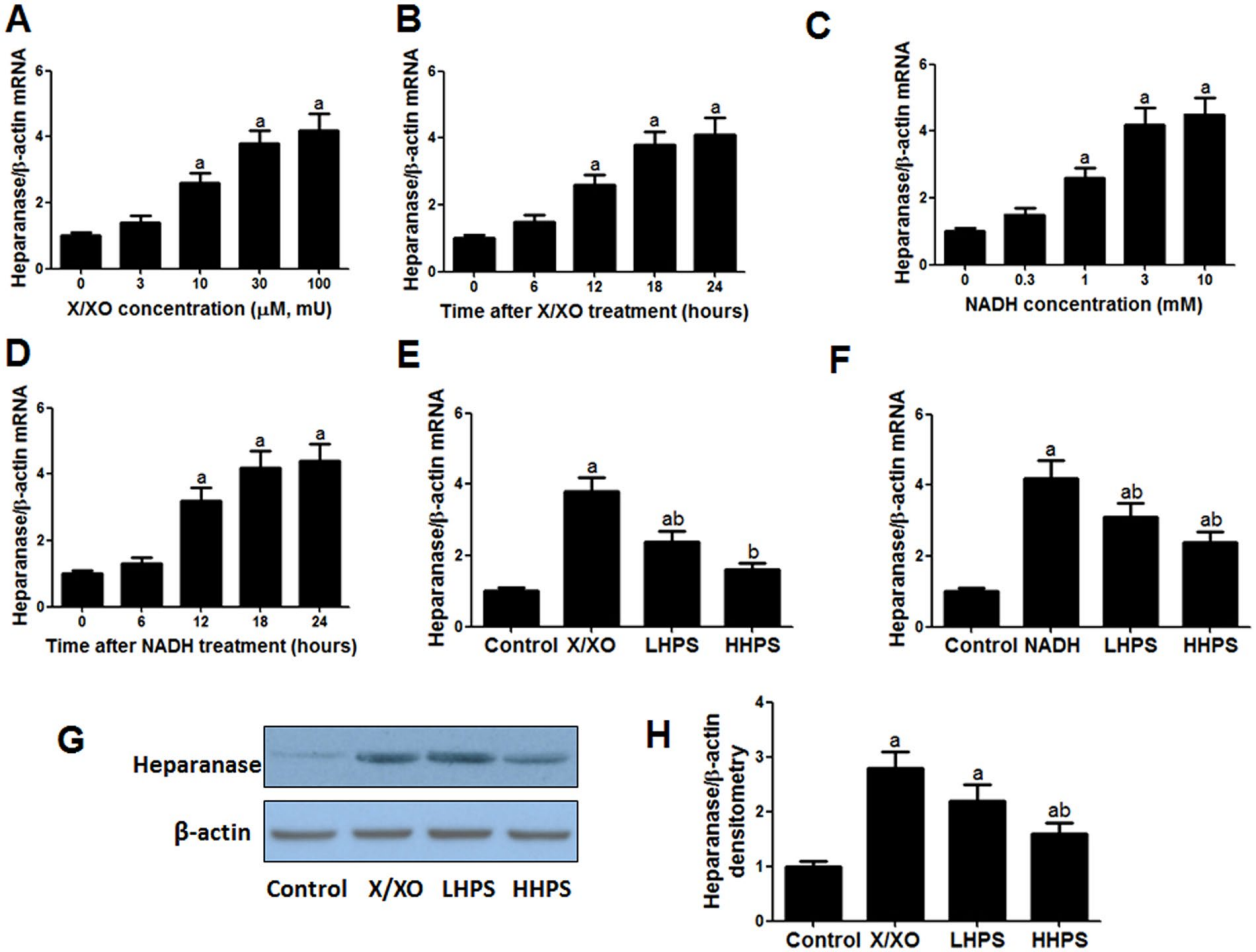
Hyperoside pre-treatment reduced the increased heparanase expression induced by ROS in cultured podocytes. (**A**) The dose-response effect of xanthine/xanthine oxidase (X/XO) on podocytes heparanase mRNA expression. Podocytes were incubated with 0, 3, 10, 30, 100 µM xanthine plus equal doses of xanthine oxidase (mU) for 18 h. (**B**) The dose-response effect of NADH on podocyte heparanase mRNA expression. Cultured podocytes were incubated with 0, 0.3, 1, 3, 10 mM NADH for 18 h. (**C**,**D**) Podocyte heparanase mRNA expression after incubation with X/XO (30 µM/mU) or NADH (3 mM) for 0, 6, 12, 18 and 24 h. (**E**) Hyperoside pre-incubation attenuated increased heparanase mRNA expression induced by extracellular ROS. Control: vehicle incubation for 18 h; X/XO: 30 µM xanthine plus 30 mU xanthine oxidase incubation for 18 h; LHPS or HHPS: 30 or 100 μg/ml hyperoside pre-incubation for 1 h and followed by X/XO (30 µM/mU) exposure. (**F**) Hyperoside pre-incubation attenuated increased heparanase mRNA expression induced by intracellular ROS. Control: vehicle incubation for 18 h; NADH: 3 mM NADH incubation for 18 h; LHPS or HHPS: 30 or 100 μg/ml hyperoside pre-incubation for 1 h and followed by NADH exposure. (**G**,**H**) Hyperoside pre-incubation attenuated heparanase protein expression induced by X/XO by western blot analysis. The columns showed the amount of heparanse protein levels relative to β-actin. Control: PBS incubation for 24 h; X/XO: X/XO (30 µM/mU) incubation for 24 h; LHPS or HHPS: 30 or 100 μg/ml hyperoside pre-incubation for 1 h and followed by X/XO (30 µM/mU) exposure. Data are presented as mean ± SD. P < 0.05 is statistically significant. ^a^Indicates significant vs. Control, ^b^indicates significant vs. ROS.

Incubation with xanthine and xanthine oxidase (X/XO) generates extracellular radicals such as superoxide and hydrogen peroxide while exposure to NADH induces intracellular generation of ROS^25^. Exposure to X/XO at concentration of 3, 10, 30 and 100 (µM/mU) or NADH (0.3, 1, 3 and 10 mM) could significantly increase podocyte heparanase mRNA levels in a dose-related manner (Fig. 5A,B). The time-response effect of X/XO (30 µM/mU) or NADH (3 mM) incubation on podocyte heparanase mRNA levels was also observed (Fig. 5C,D). The heparanase mRNA up-regulation induced by X/XO (30 µM/mU) or NADH (3 mM) could be partly or completely abolished by pre-treatment with 30 or 100 μg/ml hyperoside (Fig. 5E,F). Accordingly, the heparanase protein up-regulation induced by X/XO (30 µM/mU) could be abolished by 100 μg/ml hyperoside pre-treatment (Fig. 5G,H). Hyperoside could significantly mitigate podocyte heparanase up-regulation induced by ROS.

### ROS inhibition decreased heparanase elevation induced by high glucose

**Figure 6:**
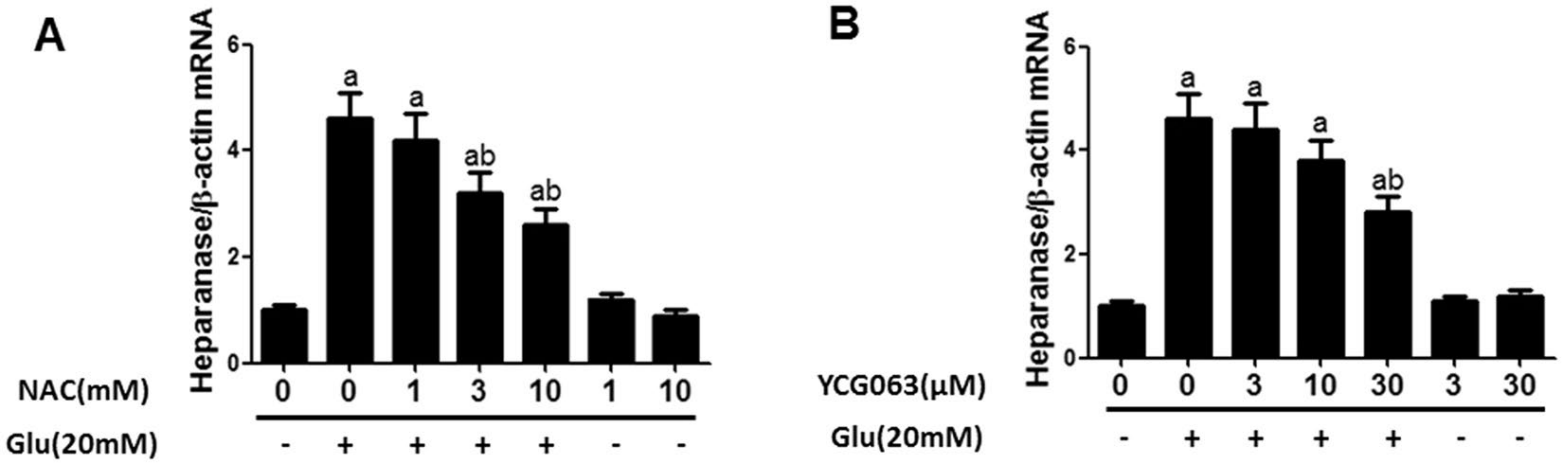
ROS inhibition blocked heparanase expression induced by high glucose stimulus in cultured podocytes. (**A**) The dose-response effect of ROS inhibitor NAC on podocyte heparanase mRNA expression. Control: PBS incubation for 18 h; Glucose: 20 mmol/L glucose incubation for 18 h; NAC + Glucose: 1, 3, 10 mM NAC pre-incubation for 1 h and followed by 20 mmol/L glucose exposure; NAC: 1, 10 mM NAC incubation alone. (**B**) The effect of ROS inhibitor YCG063 on podocyte heparanase expression. Control: PBS incubation for 18 h; Glucose: 20 mmol/L glucose incubation for 18 h; YCG063 + Glucose: 3, 10, 30 µM YCG063 pre-incubation for 1 h and followed by 20 mmol/L glucose exposure. YCG063: 3, 30 µM YCG063 incubation alone. Data are presented as mean ± SD. P < 0.05 is statistically significant. ^a^Indicates significant vs. Control, ^b^indicates significant vs. Glucose.

The dose-response effect of ROS inhibitor N-acetyl-L-cysteine (NAC) and YCG063 on heparanase expression induced by high glucose was explored. Pre-incubation with NAC (3 mM) or YCG063 (30 µM) could partly antagonize the up-regulated heparanase mRNA expression induced by 20 mmol/L glucose (P < 0.05). Incubation with NAC or YCG063 alone did not influence heparanase expression (Fig. 6A,B).

## Discussion

DN is becoming one of the life-threatening diabetic micro-vascular complications^4, 34^. The epidemical evidence showed that the incidence of DN has increased dramatically among diabetes population worldwide in recent year^4, 35^. The data from evidence-based medicine suggested that proteinuria occurred inevitably and kept increasing at the early stage of DN, despite intensive blood glucose control and RAS inhibition^36, 37^. DN often develops insidiously over many years before clinical manifestations including proteinuria and declining renal function are evident^3^. Therefore, taking precaution against the appearance of albuminuria and onset of DN is more meaningful and important than curing it in clinical practice^38^. There is a great need for alternative drugs to prevent the development of albuminuria and postpone the onset of DN in DM patients^39^.

Alteration of the permeability characteristics of GFB is an early cause for proteinuria^38, 40^. GBM damage plays an important role in GFB dysfunction and pathogenesis of DN^5, 9, 41^. The presence of glycosaminoglycans, primarily HS in GBM can prevent passage of negatively charged macromolecules cross to urinary space^7, 12^. The decreased HS content in GBM results in loss of negative selectivity at the early stage of DN^12, 16^ and then impairs the perm-selectivity to negatively charged albumin^10, 42^. Substantive albumin is leaked from plasma into urinary space and causes the formation of albuminuria in DN. The recent study showed that heparanase plays a pivotal role in the development of proteinuria in DN, which suggests that loss of HS contributes to the development of proteinuria^17, 26^.

Heparanase is the only enzyme capable of degrading HS specifically in extracellular matrix^18^. HS chains are important constituents and organizers of GBM and have a key role in maintaining the integrity and function of the glomerular filtration barrier^43^. The HPR1 gene first synthesizes a 68 kD pre-proheparanase and it is processed to 65 kD pro-heparanase in the endoplasmatic reticulum by glycosylation and cleaving off the signal peptide. Then it is transported to the Golgi apparatus, packaged into vesicles and secreted. After internalization, the 65 kD heparanase protein is then transferred to endosomes and lysosomes, where it is proteolytically into the active heterodimer consisting of an 8 kD and 50 kD subunits^18^. Heparanase is majorly up-regulated in podocytes induced by high glucose in DM status^23, 24^. Its expression could also be increased by ROS, angiotensin II and aldosterone in cultured podocytes^25, 44^ and the increased podocyte heparanase expression in kidneys has been demonstrated in DN^19, 23^, which is essential for the development of albuminuria DN in both animal model and human^26, 45^. Inhibition of heparanase expression and restoring HS contents proved to be effective to decrease proteinuria of glomerular diseases^26, 46–49^.

As an active flavone glycoside, hyperoside is documented to have therapeutic effect on ischemic reperfusion injury in brain and heart, hepatitis and cancer^50–53^. It can protect cells against oxidative stress and oxidation-related apoptosis^29, 54, 55^. Hyperoside displayed potential anti-inflammatory on high glucose induced inflammation *in vitro* and *in vivo* ^27, 28^. Our previous study showed that hyperoside has therapeutic effect on albuminuria at the early stage of DN^32^. More meaningfully, the present study offered the further experimental evidence that hyperoside has a prophylactic effect on albuminuria and GBM damage in DM mice, which could postpone the onset of DN. The results showed that orally pre-treatment with hyperoside (30 mg/kg/d) for four weeks could significantly ameliorate the albuminuria (urinary mAlb/Cr) in DM mice and slow the progression of DN. There was no significant difference in blood pressure, glucose and lipids parameters, plasma angiotensin II and aldosterone levels between DN and hyperoside pre-treatment groups. The prophylactic effect of hyperoside might not be due to its remedial effect on metabolic and hemodynamic factors. We could not completely exclude the possibility of involvement of the intra-renal RAS system which is independent of systemic RAS system because it was difficult to measure the renal tissue angiotensin II and aldosterone levels. Notably, Hyperoside could decrease ROS end product (MDA) and ROS producing enzyme (XO) and increase ROS preventing enzymes (SOD and CAT) both in kidney and circulation of DM mice, showing that it could alleviate renal oxidative stress in DM mice. The anti-oxidant property of hyperoside might partly explain its prophylactic effect on albuminuria and GBM damage in DM mice.

Consistently, transmission electron microscopy observation demonstrated that GBM thickening in DN was also alleviated by hyperoside pre-treatment. GBM thickening precedes clinically evident albuminuria and is regarded as the early pre-disposing factor of albuminuria in DN patients^5, 6^. This morphological change suggested that hyperoside had a protective effect on GBM damage in DM mice. Our results showed that hyperoside pre-treatment could restore the glomerular HS contents decreased in DN mice. Due to the major role of HS in GBM composition, its reduction was regarded as a determinant of glomerular hyper-permeability in DN^12, 16, 42^. We presumed that the preservation of HS by hyperoside might be associated with decreased heparanase function which can specifically degrade HS in GBM. With sensitive heparanase antibody that specifically interacts with the 65-kDa heparanase, we were able to detect small quantity of heparanase protein. The data manifested that renal heparanase mRNA and protein up-regulation in DM mice could be significantly improved by hyperoside pre-treatment. Our results indicated that the normalization of glomerular heparanase expression and HS content in GBM are closely associated with the protective effect of hyperoside on albuminuria development and GBM damage in DN. Admittedly, we cannot exclude the possible involvement of the other ingredients of GBM besides HS. Taken together, hyperoside pre-treatment could decrease glomerular heparanase expression and restore HS contents in GBM, displaying a protective effect on GBM damage and albuminuria in DM animal.

Because renal heparanase is mainly distributed in podocytes^23^, we speculated that hyperoside has a negative effect on podocyte heparanase expression. The *in vitro* studies showed that heparanase expression in cultured podocytes could be induced by high glucose, which is consistent with the previous study^23^. Hyperoside pre-treatment could significantly inhibit heparanase mRNA and protein up-regulation induced by high glucose. To further test whether this effect is caused by regulating heparanase promoter activation, we conducted HPR1 gene luciferase reporter gene assays in cultured podocytes. Hyperoside was found to have a direct negative effect on increased HPR1 promoter activation and heparanase transcription induced by high glucose. Certainly, we could not exclude the possibility that hyperoside could directly bind to heparanase and affect the enzyme catalytic activity of heparanase. Furthermore, whether hyperoside directly binds to the transcription regulating factor or promoters of HRP1 also remains to be deciphered.

The previous studies suggested ROS act as an important risk factor for heparanase up-regulation^25, 44^. Our data have also demonstrated that both extracellular and intracellular ROS stimulus could induce heparanase mRNA expression in podocytes. ROS-induced heparanase mRNA and protein expression in cultured podocytes could be significantly attenuated by hyperoside pre-treatment, which was coherent with our *in vivo* data. Hyperoside pre-treatment could markedly inhibit heparanase up-regualtion induced by ROS.

Oxidative damage including both free radical and non-radical oxygen species contributes to the pathogenesis of onset and progression of DN^56^. Both experimental and clinical studies have documented a close link among hyperglycemia, oxidative stress and DN^57^. High-glucose treatment increased ROS generation in podocytes^58^ and the recent study has implicated that high glucose-induced podocyte injury and apoptosis are mediated by ROS^59^. It was observed that ROS inhibitor NAC and YCG063 could partly antagonize podocyte heparanase up-regualtion induced by high glucose. Based on that, we deduced that high glucose induced heparanase up-regulation might also be partly mediated by ROS generated in podocytes. The inhibitory effect of hyperoside on podocyte heparanase expression was likely to be partly mediated by its anti-oxidative effect but needs more supportive evidence.

Although the detailed underlying molecular mechanism remains to be investigated, our current *in vitro* and *in vivo* data suggested that hyperoside pre-treatment could inhibit podocyte heparanase expression induced by high glucose and ROS and then restore HS contents in GBM in DN mice. This effect contributed to maintaining GBM composition integrity and preventing the development of albuminuria in DN. Taken together, hyperoside could be taken as a potential and promising candidate chemical compound in preventing and treatment of DN. It would be necessary to perform the translational medicine research or clinical trial upon it in the future.

## Conclusions

Our study indicated that hyperoside pre-treatment can prevent development of proteinuria and postpone the onset of DN in DM mice. The prophylactic effect of hyperoside on GBM damage in DM mice attributed to inhibiting podocyte heparanase expression induced by high glucose and oxidative stress.

## Methods

### Reagents

Hyperoside with the purity higher than 98% was purchased from Zelang Biological Technology Company (Nanjing, China) and suspended in 1% carboxymethyl cellulose (CMC) solution for animal experiments. Hyperoside (CAS#482360), N-acetyl-L-cysteine (NAC), Xanthine and xantine oxidase used for cell experiments were from Sigma Corporation (Hong Kong, China) and dissolved in 0.1% DMSO. YCG063 was bought from Calbiochem (San Diego, CA, USA) and NADH from Roche (Roche Diagnostics, Indianapolis, IN, USA).

### Diabetic nephropathy model

Type 1 diabetes was induced by streptozotocin (STZ) injection according to the Animal Models of Diabetic Complications Consortium (AMDCC) protocol with minor modification^60, 61^. Seven to eight weeks old female C57BL6 mice were given three intra-peritoneal injections of STZ (Sigma, 100 mg/kg body weight) dissolved in 10 mM sodium citrate every 48 hours. The normal mice only receiving the same volume of sodium citrate served as the control group. The mice with blood glucose between 13.9 and 22.2 mmol/L were regarded as DM mice two weeks after STZ injection. Two weeks after the DM establishment, DM mice were randomly divided into three groups: DM mice orally treated with the vehicle CMC solution alone (DN group), low or high dose of hyperoside (10 or 30 mg/kg/d, once every day) for four weeks (LHPS and HHPS groups). Each group had 15 mice and their ratio of urinary micro-albumin to creatinine (urinary mAlb/Cr) was assayed every two weeks. Those with elevated urinary mAlb/Cr (30~300 mg/g) were regarded as DN mice^60^. All animals were fed with standard laboratory diet and provided with water ad libitum.

### Experimental protocols

The experimental protocol was approved by the Animal Ethics Committee of Fudan University and all experiments were performed in accordance with relevant guidelines and regulations. Blood glucose levels of DM mice were monitored in tail vein blood every week and urine was tested for ketone bodies. When necessary, DM mice were given supportive insulin treatment (Ultratard, Novo Nordisk, Denmark) at dose of 1 IU/kg BW (twice a week) to prevent apparent exhaustion and ketosis. When experiment terminated, individual urine sample was collected and the urinary mAlb/Cr was measured and calculated according to our previous study^32^. Mean arterial pressure (MAP) was measured in conscious, trained mice at room temperature using a tail-cuff monitor (BP-2000 Blood Pressure Analysis system, Visitech Systems). Fresh kidney cortices were excised right after animals were sacrificed. Blood samples were collected and centrifuged to obtain serum. All samples were stored at −80 °C until further study.

### Enzyme-linked immunosorbent assay (ELISA)

The serum malondialdehyde (MDA) and super oxide dismutase (SOD) levels were measured by ELISA kits (Beyotime Biotechnology, Shanghai, China). The serum angiotensin II and aldosterone were also measured by commercial kits (R&D, Minneapolis, MN, USA) according to the operating instructions. MDA, SOD, catalase (CAT), xanthine oxidase (XO) levels in kidney cortices were assayed according to the manufacture (Cayman Chemical, Ann Arbor, MI, USA).

### Histological analysis

For ultrastructural evaluation, the renal cortex was cut into small pieces (1 mm^3^) and pre-fixed with 2.5% glutaraldehyde (pH = 7.4) for 48 h, then washed by 0.1 M PBS (10 min × three times). The samples were post-fixed in 1% buffered potassium osmate for 1 h, dehydrated by gradient acetone and embedded in Epon812 overnight. Ultra-thin sections were stained with 3% lead citrate/uranyl acetate and examined under transmission electron microscope (CENTRA100, Carl Zeiss, Germany). Three samples were selected from every renal cortex and each with five electron micrographs at ×25000 magnification. Five glomeruli in every electron micrograph were randomly selected for further measurement. Image-Pro Plus software was utilized to measure the mean GBM thickness and all images were analyzed by an investigator who was blinded to the identity of the samples.

### Immunofluorescence

Mice kidney cortices were fixed in 4% formalin and embedded in paraffin. Antigen retrieval and deparaffinization were performed using the antigen retrieval solution for 20 minutes at 95 °C. All sections (5 μm) were blocked with 10% bovine serum albumin (BSA) and then incubated with monoclonal anti-mouse HS primary antibody (AMS Biotechnology, Milton Park, Abingdon, UK) in the dilution of 1:100 at 4 °C overnight. The rabbit anti-mouse heparanase primary antibody (Ab85543, Abcam, Cambridge, MA) which recognizes 65 kD precursor as well as the 50 kD and 8 kD subunits of heparanase and the heparanase primary antibody (MBS711555, My Biosource) reacting with 65 kD heparanase heterodimer were adopted to display glomerular heparanase expression. The sections were washed in 0.1 M PBS and stained with FITC (or rhodamine) conjugated secondary antibodies for 60 min at room temperature. DAPI was adopted to label nuclear DNA. After washing in 0.1 M PBS for three times, the sections were imaged by a Zeiss microscope (LSM-510, Carl Zeiss, Germany). Fluorescence intensity was quantified and analyzed by National Institute of Health (NIH) Image software.

### Cell culture

Conditionally immortalized mouse podocytes were cultivated in RPMI 1640 cell medium containing 10% fetal bovine serum (FBS), 100 U/ml penicillin, 100 mg/ml streptomycin, and 2 mM L-glutamine^62^. To permit immortalized growth, the culture medium was supplemented with 10 U/ml of recombinant mouse γ-interferon to induce the expression of T antigen and cells were cultured at 33 °C (permissive conditions). To induce differentiation, cells were cultured on collagen IV under non-permissive condition (37 °C) without γ-interferon for 10–14 days^63, 64^. The differentiated podocytes were used in our cell experiments. Glucose, ROS or ROS-related inhibitors pre-treatment were performed as previous studies with minor modification^23, 25^.

### Quantitative RT-PCR

Total RNA was extracted from kidney cortices or cultured cells using RNeasy kit (Qiagen, Shanghai, China). Totally 1 μg RNA was reverse transcribed into cDNA by the Revert-Aid First Strand cDNA Synthesis Kit (Thermo Scientific, MA, USA). Heparanase expression was quantified by SYBRs Green Supermix and the ABI system (Bio-Rad Laboratories). Mouse heparanase primers: 5′-GGAGCAAACTCCGAGTGTATC-3′(forward) and 5′-CAGAATTTGACCGTTCAGTTGG-3′ (reverse). Mouse β-actin primers: 5′-AGTGTGACGTTGACATCCGTA-3′(forward) and 5′-GCCAGAGCAGTAATCTCCTTCT-3′ (reverse). Gene expression levels of heparanase were quantified by the delta-delta CT method using β-actin as the housekeeping gene. Three independent experiments were performed.

### Western blot

The mouse kidney cortices or cultured cells were homogenized in radioimmunoprecipitation assay (RIPA) buffer at 4 °C. After centrifugation (21000 × g) for 30 min at 4 °C, supernatant was collected. Total protein concentration was quantified by using the DC-protein determination system (Bio-Rad, Shanghai, China). Samples were processed for SDS-PAGE, and 30 μg protein extract were loaded and electro-transferred onto nitrocellulose membrane (Hybond-ECL, Amersham). The membranes were blocked with 5% non-fat dry milk in 1 × TBS (0.1% Tween-20) for 30 min and incubated overnight with polyclonal rabbit anti-mouse heparanase-1 antibody (MBS711555, My Biosource) which can recognize the 65 kD precursor of heparanase-1 or the monoclonal anti-mouse HS primary antibody (AMS Biotechnology, Milton Park, Abingdon, UK) in the dilution of 1:1000 at 4 °C. After incubation with horseradish peroxidase–labeled secondary antibody for 60 min, the signals were detected with an enhanced chemiluminescence system (Amersham). Membranes were incubated with rabbit polyclonal anti-β-actin antibodies (Cell Signaling Technology, USA) to serve as the internal controls for equal loading. The density of heparanase in each band was determined using NIH Image software and expressed as a relative value to the density of the corresponding β-actin band.

### Transfection and luciferase activity assay

A pGL3 luciferase reporter vector containing the 0.7 or 3.5 kb promoter region of the mouse HPR1 (heparanase) gene was constructed as the previous study^23^. The cultured podocytes in 12-well plates were treated with vehicle, Hyperoside (Sigma-Aldrich) and (or) 20 mmol/L glucose before they were transfected with either pGL3-HPR1 promoter construct or pGL3-basic empty vector. The pRL-CMV (Promega, WI, USA) containing renilla luciferase gene was adopted as an internal control for transfection efficiency using 1.5 µl Lipofectamine in 100 µl of Opti-MEM medium (Invitrogen, CA, USA). Cultured podocytes were harvested 24 h after transfection and the luciferase activity was measured by Dual-Luciferase reporter assay kit (Promega, Madison, WI).

### Statistical analysis

Data were presented as mean ± standard deviation (SD) and analyzed by Graphpad Prism 6.0 software (San Diego, CA, USA). Differences between multiple groups were analyzed by one-way ANOVA in combination with Student’s t test. The post hoc comparisons using the Student Newman–Keuls test were used for inter-group comparisons of multiple variables. P < 0.05 was considered significant.

### Ethics approval

Our experimental protocol was approved by the Animal Ethics Committee of Fudan University and all experiments were performed in accordance with relevant guidelines and regulations.

### Data availability

All the data in our manuscript are available in Jinshan Hospital of Fudan University.
